# Identification of key microRNAs and genes in preeclampsia by bioinformatics analysis

**DOI:** 10.1371/journal.pone.0178549

**Published:** 2017-06-08

**Authors:** Shouling Luo, Nannan Cao, Yao Tang, Weirong Gu

**Affiliations:** 1 The Department of Obstetrics, Obstetrics and Gynecology Hospital of Fudan University, Shanghai, China; 2 Shanghai Key Laboratory of Female Reproductive Endocrine Related Diseases, Huangpu Area, Shanghai, China; Stellenbosch University, SOUTH AFRICA

## Abstract

Preeclampsia is a leading cause of perinatal maternal–foetal mortality and morbidity. The aim of this study is to identify the key microRNAs and genes in preeclampsia and uncover their potential functions. We downloaded the miRNA expression profile of GSE84260 and the gene expression profile of GSE73374 from the Gene Expression Omnibus database. Differentially expressed miRNAs and genes were identified and compared to miRNA-target information from MiRWalk 2.0, and a total of 65 differentially expressed miRNAs (DEMIs), including 32 up-regulated miRNAs and 33 down-regulated miRNAs, and 91 differentially expressed genes (DEGs), including 83 up-regulated genes and 8 down-regulated genes, were identified. The pathway enrichment analyses of the DEMIs showed that the up-regulated DEMIs were enriched in the Hippo signalling pathway and MAPK signalling pathway, and the down-regulated DEMIs were enriched in HTLV-I infection and miRNAs in cancers. The gene ontology (GO) and Kyoto Encyclopedia of Genes and Genomes pathway (KEGG) enrichment analyses of the DEGs were performed using Multifaceted Analysis Tool for Human Transcriptome. The up-regulated DEGs were enriched in biological processes (BPs), including the response to cAMP, response to hydrogen peroxide and cell-cell adhesion mediated by integrin; no enrichment of down-regulated DEGs was identified. KEGG analysis showed that the up-regulated DEGs were enriched in the Hippo signalling pathway and pathways in cancer. A PPI network of the DEGs was constructed by using Cytoscape software, and FOS, STAT1, MMP14, ITGB1, VCAN, DUSP1, LDHA, MCL1, MET, and ZFP36 were identified as the hub genes. The current study illustrates a characteristic microRNA profile and gene profile in preeclampsia, which may contribute to the interpretation of the progression of preeclampsia and provide novel biomarkers and therapeutic targets for preeclampsia.

## Introduction

Preeclampsia (PE) is a prevalent disease characterized by hypertension and proteinuria, and it affects approximately 5%-8% of pregnancies worldwide[1]. Accumulating evidence has demonstrated that multiple genes and cellular pathways contribute to the occurrence and development of PE [2].

MicroRNAs (miRNAs) are small non-coding RNAs of approximately 19–23 nucleotides that can bind to the 3’ untranslated region of target mRNAs resulting in the degradation and translation inhibition of the mRNA, thereby regulating gene expression at the post-transcriptional level. Reportedly, up-regulated miR-210 in the placenta has been associated with the pathogenesis of PE[3], and miR-1233 might be a potential biomarker of early PE[4].

High-throughput platforms such as microarrays are increasingly valued for the analysis of miRNA and gene expression in PE. Many miRNA expression profile and gene expression profile studies on PE have been performed using microarray technology; for example, Zhu et al[5] identified 11 overexpressed microRNAs and 23 under-expressed microRNAs in PE compared to that in normal controls. Zhang et al[6] found that miR-515 family members were related to PE through the inhibition of key genes in human trophoblast differentiation. The previous studies on miRNA expression profiles in PE all had their limitations. First, all of the reported studies focused one or several of the differentially expressed miRNAs; none of them focused on the relationship between all of the differentially expressed miRNAs with PE. Second, miRbase (http://microrna.sanger.ac.uk), PicTar (http://pictar.mdc-berlin.de), TargetScan (http://www.targetscan.org) and MiRTarget2 (http://mirdb.org) were usually used to identify the target genes of the miRNAs, but the calculation principles and methods of each database are quite different, leading to a high false-positive rate. Therefore, we combined the miRNA expression profile GSE84260 with the gene expression profile GSE73374 to uncover the key miRNAs and genes that contribute to the pathology of PE and, thus, provide novel insights into potential biomarkers for PE prognosis and therapeutic strategies.

## Materials and methods

### Microarray data

The miRNA expression profile GSE84260 and the gene expression profile GSE73374 were obtained from the GEO database (http://www.ncbi.nlm.nih.gov/geo/). The GSE84260 dataset based on GPL15018 (Agilent Human miRNA V16.0 Microarray) contained 32 samples, including 16 PE placenta samples and 16 normal placenta samples. The GSE73374 dataset based on GPL16686 (Affymetrix Human Gene 2.0 ST Array) contained 36 samples, including 19 PE placenta samples and 17 normal placenta samples.

### Identification of differentially expressed miRNAs and genes and the DEMI-DEG regulatory network

Firstly, after the raw data from the miRNA profile and gene profile underwent background correction, quartile normalization and probe summarization with the limma R package [7–8], we used a classical t test to identify the miRNAs that were differentially expressed between the two groups with cutoff values |log2 FC| ≥ 1 and p values < 0.05 and to identify the genes that were differentially expressed with the cutoff values |log2FC| ≥ 0.5 and p values < 0.05. Secondly, the MiRWalk 2.0 database, which provides the largest available collection of miRNA-target interactions [9], was used to identify target genes of the differentially expressed miRNAs identified from the GSE84260 dataset. Thirdly, we downloaded the miRNA-mRNA information from the MiRWalk 2.0-validated miRNA-gene interaction information retrieval system, in which all of the genes had been identified as target genes of the miRNAs. The intersection of the target genes from the miRNA-mRNA information and the identified differentially expressed genes from the GSE73374 dataset was selected as the final set of differentially expressed genes (DEGs). Lastly, by comparing the DEGs with the miRNA-mRNA information, we were able to identify the miRNAs that target the DEGs, and those miRNAs were selected as the final differentially expressed miRNAs (DEMIs). By mapping the DEMIs and DEGs using Cytoscape (version: 3.2.0)[10], we obtained the DEMI-DEG regulatory network.

### Functional enrichment analyses of the DEMIs and DEGs

Pathway enrichment analyses of the DEMIs were performed by utilizing the in-plug clusterProfiler from the limma R package. The Gene ontology (GO), a method for annotating genes [11], and Kyoto Encyclopedia of Genes and Genomes (KEGG) pathway, which presenting the systematic analysis of gene functions[12] enrichment analyses were performed utilizing the MATHT (http://www.biocloudservice.com) to identify potential biological processes and pathways in which the DEGs are involved. P<0.05 was considered statistically significant.

### Integration of the protein-protein interaction (PPI) network

DEGs were mapped to the Search Tool for the Retrieval of Interacting Genes (STRING version: 10.0)[13], an online tool utilized to evaluate the PPI information. Interactions with a combined score > 0.4 were selected as significant. The integrated regulatory networks were constructed using the Cytoscape software.

## Results

### Identification of DEMIs and DEGs and the DEMI-DEG regulatory network

**Fig 1:**
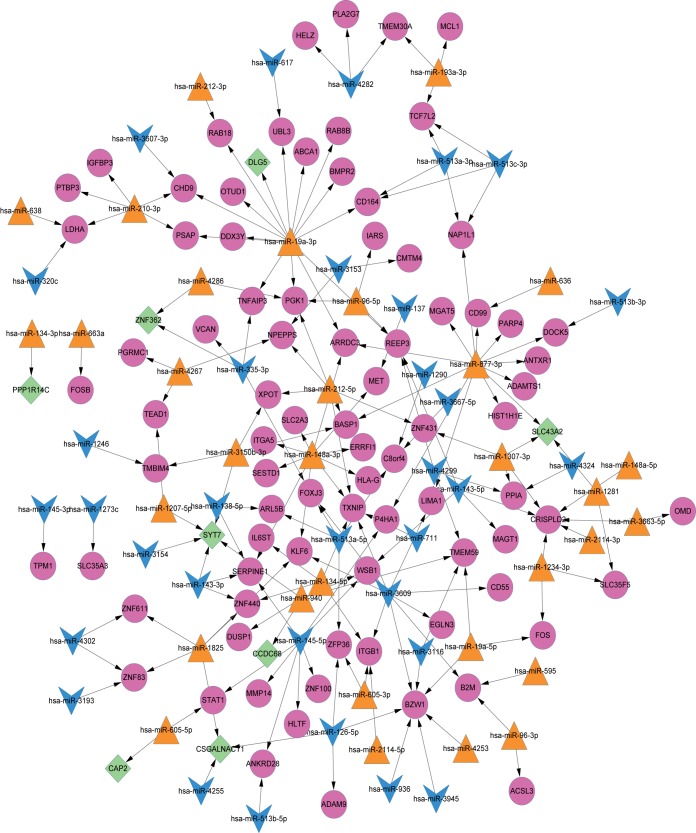
The DEMI-DEG regulatory network. Orange triangles represent up-regulated DEMIs(32); blue arrows represent the down-regulated DEMIs(33); red cycles represent the up-regulated DEGs(83); green rhombus represent the down-regulated DEGs(8).

A total of 65 differently expressed microRNAs (DEMIs), 32 up-regulated miRNAs and 33 down-regulated miRNAs, and 91 differently expressed genes (DEGs), 83 up-regulated genes and 8 down-regulated genes, were finally identified. Data for the 65 DEMIs are provided in S1 Table. In the DEMI-DEG regulatory network, there were 156 nodes and 184 interactions (Fig 1). The interaction degrees for the DEMI-DEG regulatory network represent the number of the interactions between the DEMIs and DEGs. Those DEMIs and DEGs with high interaction degrees were identified as hub nodes in the DEMI-DEG regulatory network. The top 10 DEMIs and DEGs with high degrees from the DEMI-DEG regulatory network are shown in Table 1 and Table 2.

**Table 1:** Top 10 hub DEMIs from the DEMI-DEG regulatory network.

**miRNA**	**hsa-miR-19a-3p**	**hsa-miR-877-3p**	**hsa-miR-148a-3p**	**hsa-miR-3609**	**hsa-miR-145-5p**
**Description**	upmiRNA	upmiRNA	upmiRNA	downmiRNA	downmiRNA
**degree**	15	13	10	9	8
**miRNA**	**hsa-miR-212-5p**	**hsa-miR-1825**	**hsa-miR-210-3p**	**hsa-miR-940**	**hsa-miR-134-5p**
**Description**	upmiRNA	upmiRNA	upmiRNA	upmiRNA	upmiRNA
**degree**	6	5	5	5	4

**Table 2:** Top 10 hub genes from the DEMI-DEG regulatory network.

Gene	BZW1	CRISPLD2	TXNIP	SYT7	TMEM59	SERPINE1	REEP3	PGK1	ITGB1	ZNF83
**Description**	upgene	upgene	upgene	downgene	upgene	upgene	upgene	upgene	upgene	upgene
**Degree**	7	6	5	5	4	4	4	4	4	3

### Pathway enrichment analyses of the DEMIs

**Fig 2:**
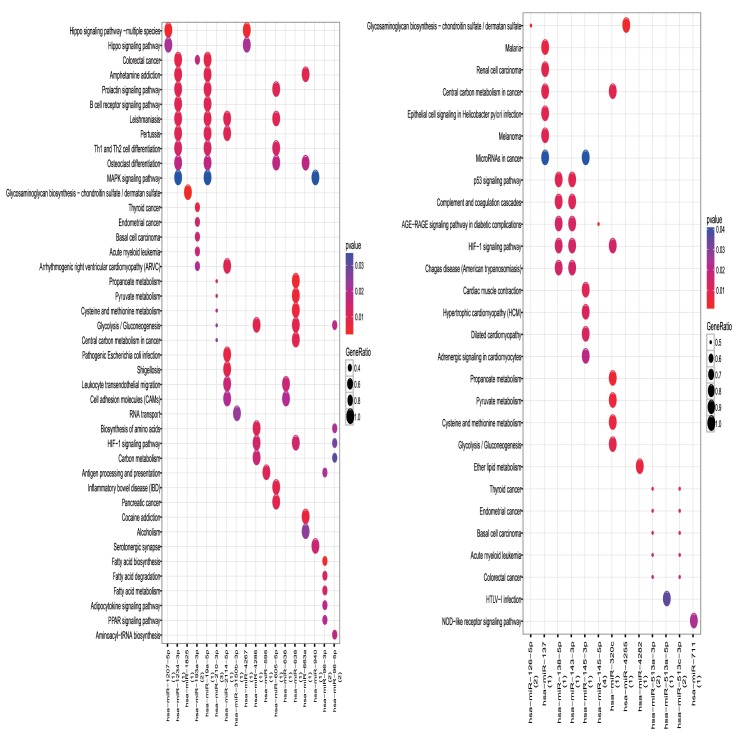
Pathway enrichment analyses of DEMIs. The left is up-regulated DEMIs and the right is down-regulated DEMIs. Red: p value is small; Blue: p value is large; the size of the bubbles means the enrichment, larger bubbles means larger generatio.

KEGG pathway analyses indicated that the up-regulated DEMIs were enriched in 150 pathways such as the Hippo signalling pathway and MAPK signalling pathway. The down-regulated DEMIs were enriched in 73 pathways such as HTLV-I infection and miRNAs in cancers (Fig 2).

### Functional enrichment analyses of the DEGs

We uploaded all DEGs to MATHT to identify the GO categories and KEGG pathways of the DEGs. The functional enrichment analysis results showed that the down-regulated DEGs (8) were not enriched in any of the categories or pathways. The GO analysis results showed that the up-regulated DEGs were mainly involved in biological processes (BP) such as the response to cAMP (Table 3). GO molecular function (MF) analysis indicated that the up-regulated DEGs were mainly involved in protein binding and growth factor binding (Table 3). In addition, for the cell component (CC) analysis, the up-regulated DEGs were significantly enriched in the extracellular exosome and membrane (Table 3). KEGG pathways analyses showed that up-regulated DEGs (83) were significantly enriched in the Hippo signalling pathway and pathways in cancer.

**Table 3:** Pathway and Gene ontology analysis of the up-regulated DEGs associated with PE (TOP5).

	ID	Name	Count	PValue
**PATHWAY**	hsa04514	Cell adhesion molecules (CAMs)	4	3.44E-02
**PATHWAY**	hsa04390	Hippo signaling pathway	4	4.02E-02
**PATHWAY**	hsa05200	Pathways in cancer	6	4.61E-02
**PATHWAY**	hsa05140	Leishmaniasis	3	4.98E-02
**PATHWAY**	hsa05412	Arrhythmogenic right ventricular cardiomyopathy (ARVC)	3	4.98E-02
**GO_BP**	GO:0051591	response to cAMP	5	6.06E-05
**GO_BP**	GO:0042542	response to hydrogen peroxide	5	9.12E-05
**GO_BP**	GO:0033631	cell-cell adhesion mediated by integrin	3	1.27E-04
**GO_BP**	GO:0071222	cellular response to lipopolysaccharide	6	1.80E-04
**GO_BP**	GO:0042493	response to drug	8	5.24E-04
**GO_CC**	GO:0070062	extracellular exosome	27	1.33E-04
**GO_CC**	GO:0016020	membrane	23	1.73E-04
**GO_CC**	GO:0005925	focal adhesion	8	1.77E-03
**GO_CC**	GO:0009897	external side of plasma membrane	6	2.60E-03
**GO_CC**	GO:0000139	Golgi membrane	8	1.60E-02
**GO_MF**	GO:0005515	protein binding	55	9.25E-04
**GO_MF**	GO:0000978	RNA polymerase II core promoter proximal region sequence-specific DNA binding	8	1.17E-03
**GO_MF**	GO:0000982	transcription factor activity, RNA polymerase II core promoter proximal region sequence-specific binding	3	4.88E-03
**GO_MF**	GO:0019838	growth factor binding	3	6.70E-03
**GO_MF**	GO:0004386	helicase activity	4	6.89E-03

### PPI network of the DEGs

**Fig 3:**
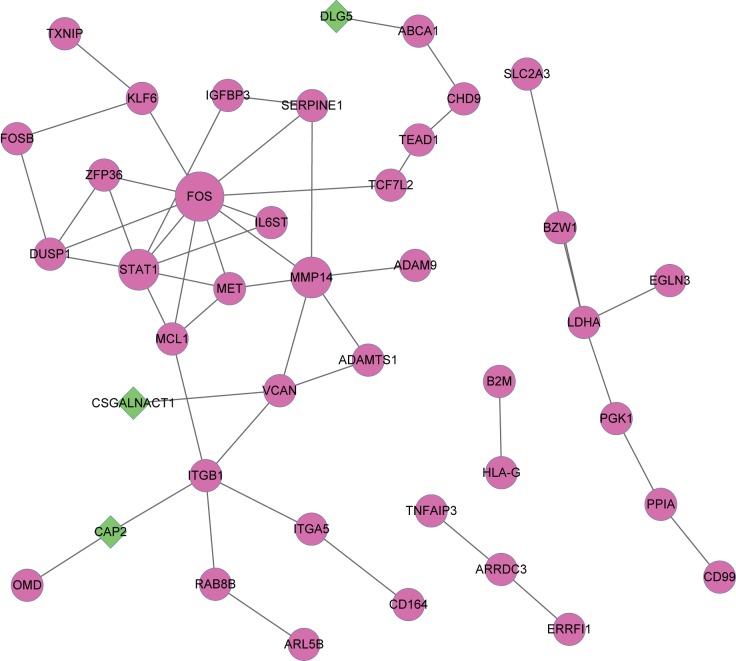
Protein-protein interaction network of DEGs. Purple cycles represent up-regulated genes, green diamonds represent down-regulated genes. The size indicates the interaction degrees.

The PPI network of the DEGs was constructed using String. In the PPI network, there were 41 nodes, including 38 up-regulated DEGs and 3 down-regulated DEGs, and 50 interactions (Fig 3). The hub nodes were FOS, STAT1, MMP14, ITGB1, VCAN, DUSP1, LDHA, MCL1, MET and ZFP36. Among all of the proteins in the PPI network, only CAP2, CSGALNACT1 and DLG5 were down-regulated.

## Discussion

PE is a multisystem disorder specific to pregnancy, and deficiency in our knowledge of the exact aetiology and pathogenesis of PE restricts the ability to treat this disease. Therefore, understanding the molecular mechanism involved in PE is extremely important to develop more effective diagnostic and therapeutic strategies. In the present study, a total of 65 DEMIs and 91 DEGs were identified. The up-regulated DEMIs were enriched in the Hippo signalling pathway and MAPK signalling pathway, and the down-regulated DEMIs were enriched in HTLV-I infection and miRNAs in cancers. FOS, STAT1, MMP14, ITGB1, VCAN, DUSP1, LDHA, MCL1, MET and ZFP36 were defined as key proteins that might provide new ideas for further studies on PE.

MiRNAs have been increasingly recognized to have a vital association with disease including PE through post-transcriptional regulation of gene expression. In the present study, miRNA expression profiles showed that miRNAs in placentas were quite different between the PE and normal group. Eight up-regulated miRNAs, including miR-19a-3p, miR-877-3p, miR-148a-3p, miR-212-5p, miR-1825, miR-210-3p, miR-940, and miR-134-5p, and two down-regulated miRNAs, miR-3609 and miR-145-5p, were identified as statistically significant different miRNAs. MiR-148a and miR-19a have been reported to influence the +3142 C/G polymorphism of HLA-G, resulting in the down-regulation of HLA-G in PE[14]. It is widely accepted that the PE syndrome consists of two successive processes, including poor placentation in early pregnancy and the following placental oxidative stress[4]. Hypoxia of the placenta is a crucial factor leading to poor biological functions of trophoblast cells. MiR-210 is a hypoxia-inducible miRNA[15] and inhibits invasion of trophoblast cells[16]. Down-regulation of miR-145 has been identified in the placenta of PE women[17]. No studies on the relationship between the other miRNAs, miR-877-3p, miR-212-5p, miR-1825, miR-940, miR-134-5p, and miR-3609, and PE have been reported. However, they are all related to the occurrence and development of carcinomas. For example, miR-212 was down-regulated in ovarian cancer, potentially due to the significant enrichment of EZH2 and H3K27me3 in the promoter region[18]. Decreased miR-940 in hepatocellular carcinoma acted as an adaptor of CXCR2 and suppressed the invasion and migration of HCC cells[19]. Considering that the conversion of the biological functions of normal cells is fundamental to the pathology of PE and carcinoma, we infer that miR-877-3p, miR-212-5p, miR-1825, miR-940, miR-134-5p, and miR-3609 might take part in the progression of PE.

KEGG pathway analysis of the DEMIs revealed that the development of PE was associated with the Hippo signalling pathway and MAPK signalling pathway. The Hippo signalling pathway could provide novel anti-cancer drug targets. Components of the Hippo signalling pathway such as Yes-associated protein 1 (YAP) and transcription regulator protein 1 (TAZ) are synergistically associated with other signalling pathways such as G protein-coupled receptor, epidermal growth factor and Wnt pathways, which play a crucial role in cell proliferation, differentiation, apoptosis, and development[20]. Recent evidence indicates that the p38 MAPK signalling pathway is one of the key pathways in vascular endothelial cell dysfunction in PE. Activated p38 MAPK in the placenta of PE could significantly increase the levels of sEng and sFlt-l in maternal serum[21]. Gadd45α(DNA damage-inducible 45 alpha) is an oxidative stress-induced factor with high levels in PE. Gadd45αinhibits trophoblast invasion and regulates anti-angiogenesis factor secretion via the p38 MAPK signalling pathway[22].

GO and KEGG pathway analyses were performed to better understand the interactions of the DEGs. The GO analyses showed that up-regulated DEGs were intensively involved in the BP of the response to cAMP, response to hydrogen peroxide and cell-cell adhesion mediated by integrin. Furthermore, the KEGG pathways of the up-regulated DEGs included the Hippo signalling pathway and pathways in cancer. The hub genes with top degrees in the PPI network were FOS, STAT1, MMP14, ITGB1, VCAN, DUSP1, LDHA, MCL1, MET, and ZFP36. FOS was identified as up-regulated in PE, which was consist with that reported by Song[23]. FOS is involved in the regulation of angiogenesis by encoding the transcription factor c-fos proto-oncogene[24]. The second hub gene, STAT1 (signal transducers and activators of transcription 1), is phosphorylated, forming a dimer that activates Janus tyrosine kinases (JAKs) when initiated by IFN-γ[25]. Endothelial activation and excessive inflammation are the characteristics of PE, which can be induced by the IFN-γ/STAT1 signalling pathway[26]. It has been proposed that the mouse systolic arterial pressure and plasma levels of sEng were increased compared to those exposed to doxycycline, a compound that could block the transcription of MMP-14. sEng and sFlt-1, contributing to the maternal vascular dysfunction, while up-regulated MMP14 released by endothelial cells induced the release of sEng and sFlt-1[27]. Previous studies have reported that LDHA was up-regulated in PE[28–29]. Activated by hypoxia, the LDH isozyme in trophoblasts can induce higher lactate production[30], which might inhibit germ cell death dose-dependently in the human testis[31]. The MKP/DUSPs family acts as negative feedback regulators of MAPK activity by dephosphorylating phosphorylated tyrosine or serine/threonine[32]. Christe et al. reported that DUSP9/MKP-4 was essential for placental function [33]. DUSP5 may mediate the H19 down-regulation-induced suppression of proliferation and apoptosis of JAR cells [34]. The other five hub genes are ITGB1, MCL1, VCAN, MET and ZFP36. Two previous studies have reported that ITGB1 is related to PE through encoding the beta subunit of integrin. Additionally, up-regulated miR-29b might contribute to PE via its target genes ITGB1 and MCL1[35–36]. The Mtd/Mcl-1 system plays a crucial role in regulating trophoblast cell functions in both physiological and pathological conditions; Mcl-1 induces apoptosis and reduced proliferation, while Mtd likely shows different properties[37]. In preeclampsia, the Mtd/Mcl-1 system is altered towards the production of killer isoforms, meaning that both Mtd-L and Mtd-P were increased, and the expression of Mcl-1 was down-regulated in PE[38–39]. Further studies are needed to identify the functions of ITGB1 and MCL1 in PE. No studies on VCAN in PE have been reported. Met, an anti-angiogenic factor was significantly elevated in both the second and third trimesters of PE[40], but Zeng found that the plasma sMet concentration was significantly lower in women with severe PE than in control groups [41]. ZFP36 is a zinc-finger protein and can regulate the production of growth factors and cytokines by destabilizing mRNAs. Recently, one study found that ZFP36 might be a potential regulator of VEGF to control reepithelialization and angiogenesis in the skin[42].

In conclusion, we identified several abnormally expressed miRNAs and genes in PE that may participate in the pathogenesis of PE. Our study provides a comprehensive bioinformatic analysis of DEMIs and DEGs in PE, helps to understand the underlying molecular mechanisms of PE, and may provide potential biomarkers and therapeutic targets for PE. Further experiments are required to confirm the expression and potential functions of the identified miRNAs and genes in PE.

## Supporting information

S1 Table Thirty-three up-regulated DEMsI and thirty-two down-regulated DEMIs in the DEMI-DEG regulatory network. (DOCX)
