# Supplementary material for: Identification of key microRNAs and genes in preeclampsia by bioinformatics analysis
Source: PLoS One. 2017 Jun 8;12(6):e0178549. doi: 10.1371/journal.pone.0178549 (PMC5464566; doi:10.1371/journal.pone.0178549)
Supplement: S1 Table — (DOCX) [file pone.0178549.s001.docx]

#### S1 Table Thirty-three up-regulated DEMsI and thirty-two down-regulated DEMIs in the DEMI-DEG regulatory network

| name | description | degree | name | description | | degree |
| --- | --- | --- | --- | --- | --- | --- |
| hsa-miR-3609 | downmiRNA | 9 | hsa-miR-19a-3p | | upmiRNA | 15 |
| hsa-miR-145-5p | downmiRNA | 8 | hsa-miR-877-3p | | upmiRNA | 13 |
| hsa-miR-335-3p | downmiRNA | 4 | hsa-miR-148a-3p | | upmiRNA | 10 |
| hsa-miR-143-5p | downmiRNA | 4 | hsa-miR-212-5p | | upmiRNA | 6 |
| hsa-miR-513a-5p | downmiRNA | 4 | hsa-miR-1825 | | upmiRNA | 5 |
| hsa-miR-126-5p | downmiRNA | 4 | hsa-miR-210-3p | | upmiRNA | 5 |
| hsa-miR-143-3p | downmiRNA | 3 | hsa-miR-940 | | upmiRNA | 5 |
| hsa-miR-4299 | downmiRNA | 3 | hsa-miR-134-5p | | upmiRNA | 4 |
| hsa-miR-4282 | downmiRNA | 3 | hsa-miR-1307-3p | | upmiRNA | 4 |
| hsa-miR-513c-3p | downmiRNA | 3 | hsa-miR-3150b-3p | | upmiRNA | 4 |
| hsa-miR-513a-3p | downmiRNA | 3 | hsa-miR-4267 | | upmiRNA | 3 |
| hsa-miR-4302 | downmiRNA | 2 | hsa-miR-193a-3p | | upmiRNA | 3 |
| hsa-miR-3153 | downmiRNA | 2 | hsa-miR-96-5p | | upmiRNA | 3 |
| hsa-miR-3116 | downmiRNA | 2 | hsa-miR-1234-3p | | upmiRNA | 3 |
| hsa-miR-138-5p | downmiRNA | 2 | hsa-miR-19a-5p | | upmiRNA | 3 |
| hsa-miR-3193 | downmiRNA | 1 | hsa-miR-1207-5p | | upmiRNA | 2 |
| hsa-miR-617 | downmiRNA | 1 | hsa-miR-1281 | | upmiRNA | 2 |
| hsa-miR-711 | downmiRNA | 1 | hsa-miR-4286 | | upmiRNA | 2 |
| hsa-miR-145-3p | downmiRNA | 1 | hsa-miR-605-3p | | upmiRNA | 2 |
| hsa-miR-1246 | downmiRNA | 1 | hsa-miR-3663-5p | | upmiRNA | 2 |
| hsa-miR-3154 | downmiRNA | 1 | hsa-miR-605-5p | | upmiRNA | 2 |
| hsa-miR-1273c | downmiRNA | 1 | hsa-miR-96-3p | | upmiRNA | 2 |
| hsa-miR-4324 | downmiRNA | 1 | hsa-miR-212-3p | | upmiRNA | 1 |
| hsa-miR-137 | downmiRNA | 1 | hsa-miR-134-3p | | upmiRNA | 1 |
| hsa-miR-320c | downmiRNA | 1 | hsa-miR-638 | | upmiRNA | 1 |
| hsa-miR-513b-3p | downmiRNA | 1 | hsa-miR-2114-5p | | upmiRNA | 1 |
| hsa-miR-4255 | downmiRNA | 1 | hsa-miR-663a | | upmiRNA | 1 |
| hsa-miR-3607-3p | downmiRNA | 1 | hsa-miR-148a-5p | | upmiRNA | 1 |
| hsa-miR-1290 | downmiRNA | 1 | hsa-miR-2114-3p | | upmiRNA | 1 |
| hsa-miR-3667-5p | downmiRNA | 1 | hsa-miR-636 | | upmiRNA | 1 |
| hsa-miR-3945 | downmiRNA | 1 | hsa-miR-4253 | | upmiRNA | 1 |
| hsa-miR-936 | downmiRNA | 1 | hsa-miR-595 | | upmiRNA | 1 |
| hsa-miR-513b-5p | downmiRNA | 1 |  | |  |  |
